# Comparative Analysis of the Chloroplast Genome for Four *Pennisetum* Species: Molecular Structure and Phylogenetic Relationships

**DOI:** 10.3389/fgene.2021.687844

**Published:** 2021-07-27

**Authors:** Jin Xu, Chen Liu, Yun Song, Mingfu Li

**Affiliations:** Institute of Plant Inspection and Quarantine, Chinese Academy of Inspection and Quarantine, Beijing, China

**Keywords:** chloroplast genome, phylogeny, comparative analysis, *Pennisetum* species, important pasture

## Abstract

The genus *Pennisetum* (Poaceae) is both a forage crop and staple food crop in the tropics. In this study, we obtained chloroplast genome sequences of four species of *Pennisetum* (*P. alopecuroides*, *P. clandestinum*, *P. glaucum*, and *P. polystachion*) using Illumina sequencing. These chloroplast genomes have circular structures of 136,346–138,119 bp, including a large single-copy region (LSC, 79,380–81,186 bp), a small single-copy region (SSC, 12,212–12,409 bp), and a pair of inverted repeat regions (IRs, 22,284–22,372 bp). The overall GC content of these chloroplast genomes was 38.6–38.7%. The complete chloroplast genomes contained 110 different genes, including 76 protein-coding genes, 30 transfer RNA (tRNA) genes, and four ribosomal RNA (rRNA) genes. Comparative analysis of nucleotide variability identified nine intergenic spacer regions (*psbA-matK, matK-rps16, trnN-trnT, trnY-trnD-psbM, petN-trnC, rbcL-psaI, petA-psbJ, psbE-petL*, and *rpl32-trnL*), which may be used as potential DNA barcodes in future species identification and evolutionary analysis of *Pennisetum*. The phylogenetic analysis revealed a close relationship between *P. polystachion* and *P. glaucum*, followed by *P. clandestinum* and *P. alopecuroides*. The completed genomes of this study will help facilitate future research on the phylogenetic relationships and evolution of *Pennisetum* species.

## Introduction

The genus *Pennisetum* L. Rich. belongs to tribe Paniceae of the family Poaceae; it consists of approximately 140 species that are widely distributed in the tropical and subtropical zone regions all over the world. *Pennisetum* which is divided into three sections Sect. *Gymnothrix*, Sect. *Penicillaria* and Sect. *Pennisetum* is one of the largest genera in the tribe Paniceae (Pilger, 1940; DeLisle, 1963; Brunken, 1977). *Pennisetum* is an economically important grain crop that is widely cultivated in Africa and Asia (*P. glaucum*), as well as a pasture crop (*P. purpureum*, *P. ramosum*, *P. orientale*, and *P. clandestine*) and ornamental plant (*P. villosum* and *P. setaceum*) (Martel et al., 1997; Abdi et al., 2019). Many of these species are climate resilient (Kumar et al., 2019), adapted to low-fertility soils, exhibit strong stress resistance, possess high ornamental value, and are perennial in habit. Consequently, *Pennisetum* (Poaceae) species have become increasingly visible in many countries. This genus is a heterogeneous assemblage of species with different reproductive behaviors (sexual or apomictic), chromosome numbers (x = 5, 7, 8, and 9), ploidy levels (diploid to octoploid), and life cycles (annual, biennial, or perennial) (Martel et al., 1997). The classification and identification of the genus *Pennisetum* remains a challenge. *Pennisetum* and *Cenchrus* are closely related genera of Paniceae, both distributed in tropical and sub-tropical regions. The distinction between *Pennisetum* and *Cenchrus* is not clearly defined, and several species that are now included in *Cenchrus* have previously been assigned to *Pennisetum* (Donaldio et al., 2009; Chemisquy et al., 2010). Previous studies of *Pennisetum* have focused on isozymic classification, genetic diversity (Tostain, 1994; Zhang et al., 2016b), and chromosomes (Martel et al., 2004; Techio et al., 2010; Zhang et al., 2015). However, these markers have low variation. Therefore, there is a need to develop effective genetic markers to facilitate the identification, conservation, utilization, and breeding of *Pennisetum* species.

The chloroplast is a unique organelle that is also the main site of photosynthesis, effectively turning the light of the sun into chemical energy in higher plants and some algae. It has its own independent genome that ranges in size from 120 to 180 kb in higher plants (Li C. et al., 2020; Li D. et al., 2020). It has a simple structure with a low molecular weight and multiple copies (Huang et al., 2020). The structure of the chloroplast genome is circular with quadripartite organization, including a small single-copy region (SSC), a large single-copy region (LSC), and a pair of inverted regions (IRs) (Kim and Lee, 2004; Jansen et al., 2005; Yang et al., 2010; Schwarz et al., 2015; Wang et al., 2016; Mader et al., 2018; Meng et al., 2018). In 1986, the first chloroplast genomes were reported from liverwort (Ohyama et al., 1986) and tobacco (Shinozaki et al., 1986). With the rapid development of next-generation sequencing technologies, such as Roche/454 GS FLX and Illumina GenomeAnalyzer, chloroplast genome sequences can be more efficiently and economically obtained. Approximately 4,100 plant chloroplast genomes have been published and are available in the NCBI database^1^. The chloroplast genome contains a large number of functional genes, and their application value in the study of species identification and phyletic evolution has gradually been widely accepted by researchers (Kim and Lee, 2004; Raubeson et al., 2007; Wang et al., 2008, 2016; Ma et al., 2017; Chen et al., 2018; Fan et al., 2018).

In this study, we completely sequenced and compared the chloroplast genome of four species of *Pennisetum* (*P. alopecuroides*, *P. clandestinum*, *P. glaucum*, and *P. polystachion*) based on next-generation sequencing methods. Our goal was to expand our understanding of the genetic divergence of *Pennisetum* and identify potential DNA barcodes for identifying *Pennisetum* species. This study also presents the first sequenced member of the genus *Pennisetum.* The results will provide basic data for molecular phylogenetic and evolutionary research at the species level.

## Materials and Methods

### Plant Materials and DNA Extraction

Seeds of *P.alopecuroides* (Sect. *Gymnothrix*), *P. glaucum* (Sect. *Penicillaria*),*P.polystachion* (Sect. *Pennisetum*), and *P.clandestinum* (Sect. *Pennisetum*) were acquired from the national medium term genebank of perennial herbage germplasm resources hosted by the Institute of Grassland Research of Chinese Academy of Agricultural Sciences and Professor Li from the National Animal Husbandry Station, under accession number of 01068, 08, HN495, and HN294, respectively. The samples were planted in the laboratory under suitable conditions at the Chinese Academy of Inspection and Quarantine until leaves appeared on the plants. Voucher specimens of each collected species were deposited at the Institute of Plant Quarantine, Chinese Academy of Inspection and Quarantine (Voucher No.2019099PA01, 2019099PG01, 2019099PP01, and 2019099PC01). Total genomic DNA was extracted from the fresh leaf of a single plant using the method of Li et al. (2013). The total DNA quantity was evaluated on 1.0% (w/v) agarose gel electrophoresis SYBR Green I, which can assess the concentration and purity of the DNA samples.

### Illumina Sequencing and Assembly

Paired-end (PE) libraries of 350-bp insert sizes were constructed using the Nextera XT DNA library Prep Kit (Illumina Inc., San Diego, CA, United States) according to the manufacturer’s instructions. The Illumina HiSeq X-ten platform was used to perform sequencing, with PE 150-bp reads for each sample.

Raw reads were trimmed using Trimmomatic v0.32, and the resulting clean data were used for assembly and subsequent analysis (Bolger et al., 2014). The clean reads were then assembled into contigs using SPAdes 3.6.1 (Bankevich et al., 2012) with different K-mer parameters. The resulting contigs were selected for chloroplast genome-encoding contigs on BLAST searches. The selected contigs were secondarily assembled using Sequencher 5.4.5 (Gene Codes, Ann Arbor, MI).

### Annotation and Comparative Analysis

The assembled chloroplast genome annotation was performed with Plann (Daisie and Quentin, 2015), and the *P. glaucum* chloroplast genome was used as the reference. The circular chloroplast genome map with structural features was developed using the OrganellarGenomeDRAW (OGDRAW) software (Stephan et al., 2019). CodonW software (Sharp and Li, 1987) was adopted to analyze the relative synonymous codon usage (RSCU). The assembled chloroplast genome sequences of four *Pennisetum* species were deposited in NCBI under the Genbank accession number MN180104, MW816925-MW816927. Comparative analysis of the complete chloroplast genomes of four *Pennisetum* species was performed using the mVISTA program (Kelly et al., 2004).

### Analysis of Tandem and Simple Sequence Repeats (SSRs)

GMATA (Wang and Wang, 2016) software was used to detect the chloroplast SSRs in the four *Pennisetum* chloroplast genome sequences, in which the minimum numbers of repeats for mononucleotide, dinucleotides, trinucleotides, tetranucleotides, pentanucleotide, and hexanucleotides were 10, five, four, three, three, and three, respectively. Five types of repeat sequences, namely forward, reverse, complementary, palindromic, and tandem repeats, were identified in the *Pennisetum* chloroplast genome. Forward, reverse, palindrome, and complementary sequences were identified as described by running the REPuter program (Kurtz et al., 2001) with a minimum repeat size of 30 bp and similarities of 90% or greater sequence identity. Tandem repeats were identified using Tandem Repeats Finder^2^, with alignment parameters being set to two, seven, and seven for matches, mismatches, and indels, respectively. At the same time, the SSRs of the LSC, SSC, IR, and coding regions, introns, and intergenic regions that correspond to different regions were analyzed.

### Sequence Divergence Analysis

The sequences of the *Pennisetum* chloroplast genomes were aligned using MAFFT v7 (Katoh and Standley, 2013), following which the alignments were inspected and manually adjusted using Se-Al 2.0 (Rambaut, 1996). MEGA 7.0 software was used to calculate the variable and parsimony-informative base sites and the k2p-distances among the chloroplast genomes (Sudhir et al., 2016).

To identify rapidly evolving molecular markers that can be used in further *Pennisetum* phylogenetic studies, four *Pennisetum* whole genomes were included in the comparisons to perform sliding window analysis and evaluate the nucleotide variability (Pi) among chloroplast genomes using DnaSP v5.10 software (Librado and Rozas, 2009). The step size was set to 200 bp, and the window length was set to 800 bp.

### Phylogenetic Analysis

In this study, a total of 32 species, including 31 from Panicoideae and one outgroup species (*Limnopoa meeboldii*), were used for assessing phylogenetic relationships by constructing a maximum likelihood tree based on the sequences of 76 protein-coding genes. Of these, 28 chloroplast genomes were downloaded from the NCBI database. The sequences of 76 shared protein-coding genes were extracted from the GenBank formatted file containing all chloroplast genomes using Geneious v11, and alignments of genes were performed using MAFFT v7 (Kearse et al., 2012; Katoh and Standley, 2013), following which they were manually adjusted using Se-Al 2.0 (Rambaut, 1996) as needed. Finally, the data for the chloroplast genome sequences were used to perform phylogenetic analysis among the *Pennisetum* species.

The concatenated data were analyzed using the maximum likelihood (ML) and the Bayesian inference (BI) methodologies. Prior to ML and BI analyses, the best-fit model of sequence evolution, namely a general time reversible and gamma distribution (GTR + G) model, was selected using ModelFinder v.1.6 (Kalyaanamoorthy et al., 2017) under the Akaike Information Criterion. The ML analyses were performed using RAxML v.8.1 (Stamatakis, 2014) with 1,000 bootstrap replicates. The BI analysis was conducted using MrBayes v.3.2 (Fredrik et al., 2012) using the CIPRES Science Gateway. The Markov chain Monte Carlo (MCMC) algorithm was run for 2 × 5,000,000 generations, with trees sampled every 1,000 generations and the first 25% of generations discarded as burn-in. The remaining trees were used to construct a 50% majority rule consensus tree and estimate posterior probabilities. Posterior probabilities (PP) > 0.95 were considered significant support for a clade.

## Results

### Chloroplast Genome Organization of *Pennisetum*

**FIGURE 1:**
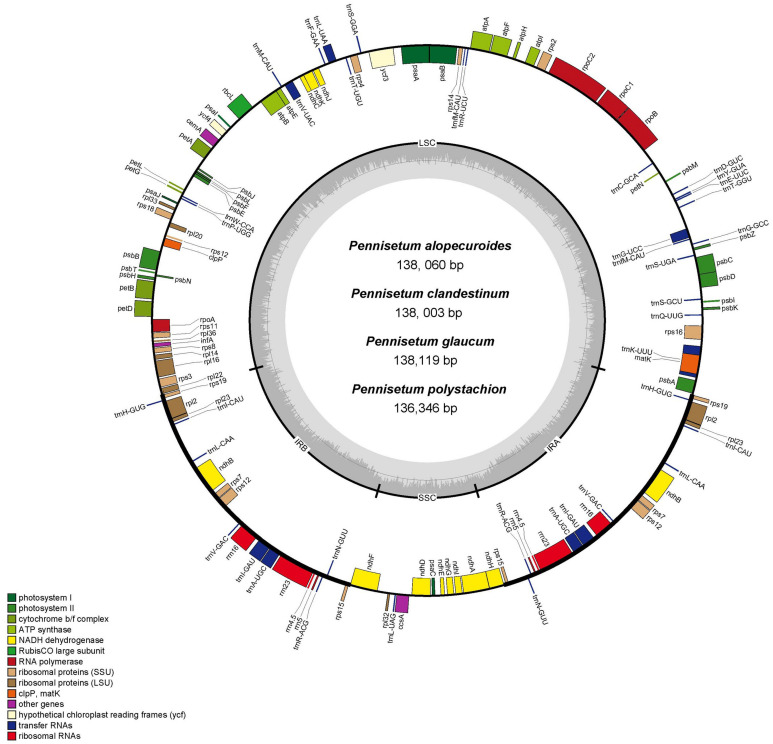
Gene map of the complete chloroplast genome of four *Pennisetum* species. Genes inside the circle are transcribed clockwise, and those outside are transcribed counterclockwise. The different colors of the blocks represent different functional groups. The darker gray color of the inner circle corresponds to the GC content, and the lighter gray color corresponds to the AT content.

In total, we obtained the chloroplast genomes of four *Pennisetum* species using the Illumina HiSeq X-ten platform. The coverage of four species were 697 (*P. polystachion*), 472 (*P. glaucum*), 230 bp (*P. alopecuroides*), and 1635 (*P. clandestinum*), respectively (Supplementary Table 1). All four species of *Pennisetum* that were sequenced had a typical double-stranded circular DNA molecule with a quadripartite structure. The assembled chloroplast genomes were 136,346 bp (*P. polystachion*), 138,119 bp (*P. glaucum*), 138,060 bp (*P. alopecuroides*), and 138,003 bp (*P. clandestinum*) in length. They consisted of a pair of IR regions (22,284–22,372 bp) separated by LSC (79,380–81,186 bp) and SSC (12,212–12,409 bp) regions (Figure 1 and Table 1). The size of the *P. glaucum* chloroplast genome (138,119 bp) was the largest among the four sequenced *Pennisetum* species, being 1,773 bp longer than that of *P. polystachion*, 59 bp longer than that of *P. alopecuroides*, and 116 bp longer than that of *P. clandestinum*. The total Guanine-Cytosine (GC) content was nearly identical in the chloroplast genomes of the four *Pennisetum* species. The GC content of both *P. alopecuroides* and *P. glaucum* was 38.6%, and that of both *P. clandestinum* and *P. polystachion* was 38.7%. Furthermore, the GC content in the IR regions (44.0–44.1%) was noticeably higher than that in the SSC (32.9–33.2%) and LSC (36.5–36.6%) regions in each chloroplast genome.

**TABLE 1:** Summary statistics for the assembly of the chloroplast genomes of four *Pennisetum* species.

	***P. alopecuroides***	***P. clandestinum***	***P. glaucum***	***P. polystachion***
Genome size (bp)	138060	138003	138119	136346
LSC length (bp)	81186	80852	81034	79380
SSC length (bp)	12212	12407	12409	12398
IR length (bp)	22331	22372	22338	22284
LSC GC content (%)	36.5%	36.5%	36.5%	36.6%
SSC GC content (%)	33.2%	33.0%	33.0%	32.9%
IR GC content (%)	44.1%	44.1%	44.0%	44.1%
Total GC content (%)	38.6%	38.7%	38.6%	38.7%
Total number of genes	110	110	110	110
Protein coding genes	76	76	76	76
rRNA	4	4	4	4
tRNA	30	30	30	30

Each of the analyzed chloroplast genomes contained a total of 110 unique genes, including 76 protein-coding genes, four ribosomal RNA (rRNA) genes, and 30 transfer RNA (tRNA) genes (Table 1). Among them, 19 of these genes were duplicated in the IR regions and contained seven protein-coding genes (*rps15*,*rps12*, *rps7*, *ndhB*, *rpl23*, *rpl2*, and *rps19*), eight tRNA genes (*trnN-GUU*, *trnA-UGC*, *trnI-GAU*, *trnV-GAC*, *trnL-CAA*, *trnI-CAU*, and *trnH-GUG*), and four rRNA genes (*rrn23*, *rrn5*, *rrn4.5*, *rrn16*). In total, 16 genes (*ycf3*, *atpF*, *petB*, *petD*, *ndhA*, *ndhB*, *rps12*, *rpl2*, *rpl16*, *trnA-UGC*, *trnG-GCC*, *trnI-GAU*, *trnK-UUU*, *trnL-UAA*, *trnV-UAC*, and *clpP*) with one intron were found (Table 2). Among the 16 intron containing genes, four genes (*trnA-UGC*, *trnI-GAU*, *ndhB*, and *rpl2*) occurred in both IR regions, one gene (*ndhA*) was in the SSC region, 10 genes (*ycf3*, *atpF*, *petB*, *petD*, *rpl16*, *trnG-GCC*, *trnK-UUU*, *trnL-UAA*, *trnV-UAC*, and *clpP*) were located in the LSC region, and one gene (*rps12*) is a trans-spliced gene with a first exon that was located in the LSC region and the other two exons located in both IR regions. The *matK* was located within the intron of *trnK-UUU*.

**TABLE 2:** List of annotated genes in the chloroplast genome of *P. glaucum.*

**Category for genes**	**Group of gene**	**Name of gene**
Photosynthesis related genes	Rubisco	*rbcL*
	Photosystem I	*psaA, psaB, psaC, psaI, psaJ*
	Assembly/stability of photosystem I	*ycf3, ycf4
	Photosystem II	*psbA, psbB, psbC, psbD, psbE, psbF, psbH, psbI, psbJ, psbK, psbL, psbM, psbN, psbT, psbZ*
	ATP synthase	atpA, atpB, atpE, *atpF, atpH, atpI
	Cytochrome b/fcompelx	petA, *petB, *petD, petG, petL, petN
	Cytochrome c synthesis	*ccsA*
	NADPH dehydrogenase	*ndhA, *ndhB, ndhC, ndhD, ndhE, ndhF, ndhG, ndhH, ndhI, ndhJ, ndhK
Transcription and translation related genes	Transcription	*rpoA, rpoB, rpoC1, rpoC2*
	Ribosomal proteins	rps2, rps3, rps4, rps7, rps8, rps11, *rps12, rps14, rps15, rps16, rps18, rps19, *rpl2, rpl14, *rpl16, rpl20, rpl22, rpl23, rpl32, rpl33, rpl36
	Translation initiation factor	*infA*
RNA genes	Ribosomal RNA	*rrn5, rrn4. 5, rrn16, rrn23*
	Transfer RNA	*trnA-UGC, trnC-GCA, trnD-GUC, trnE-UUC, trnF-GAA, trnG-UCC, *trnG-GCC, trnH-GUG, trnI-CAU, *trnI-GAU, *trnK-UUU, trnL-CAA, *trnL-UAA, trnL-UAG, trnfM-CAUI, trnM-CAU, trnN-GUU, trnP-UGG, trnQ-UUG, trnR-ACG, trnR-UCU, trnS-GCU, trnS-GGA, trnS-UGA, trnT-GGU, trnT-UGU, trnV-GAC, *trnV-UAC, trnW-CCA, trnY-GUA
Other genes	RNA processing	*matK*
	Carbon metabolism	*cemA*
	Proteolysis	*clpP

Intron-containing genes are marked by asterisks (*).

### Comparative Analysis of the Chloroplast Genome

**FIGURE 2:**
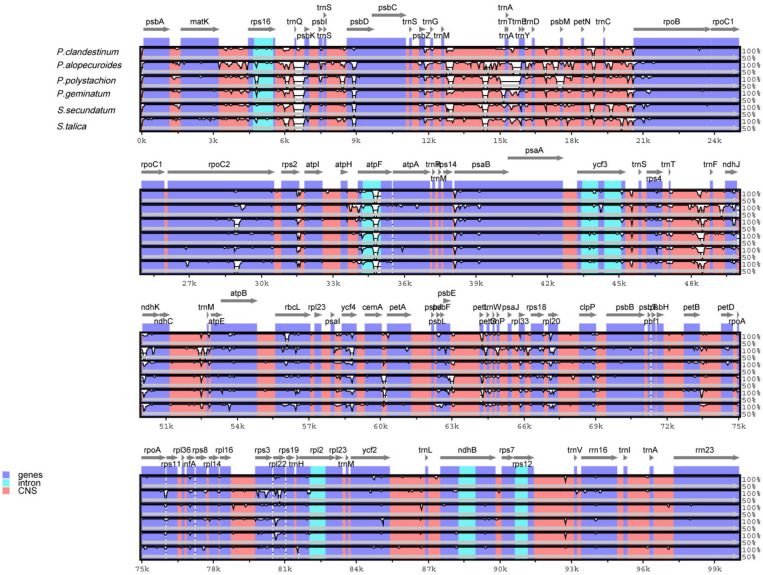
Comparison of chloroplast genomes of four *Pennisetum* species and other three *Paniceae* species (*P. geminatum*, *S. secundatum*, and *S. talica*) with *P. glaucum* as the reference using mVISTA program. The top line shows the genes in order. A cut-off of 70% identity was used for the plots and the *Y*-scale represents the percent identity between 50 and 100%.

In this study, the annotated *P. glaucum* chloroplast genome was used as a reference in mVISTA for the alignment of the chloroplast genome among the four *Pennisetum* species and three Pancoideae species (Figure 2). The mVISTA-based identity plot showed conservation in DNA sequence and gene synteny with the whole chloroplast genome and revealed the regions with increased genetic variation. The comparison evidently showed considerable similarities in genome composition and size among the seven species. These species shows a closer relationship because they belongs to the same subfamily Panicoideae. Furthermore, the Pi value was calculated for the chloroplast genome sequences. A total of 139,958 sites, including 1,887 variable sites (1.35%) and 224 parsimony-informative sites (0.16%), were detected across the complete chloroplast genomes (Table 3). We found that the LSC and SSC regions were more variable than the two IR regions, with average Pi values of 0.00161 in the IR regions, 0.00914 in the LSC regions, and 0.01111 in SSC regions. The non-coding region was more variable than the coding region, and the intergenic space was the most variable in the chloroplast genomes.

**TABLE 3:** Variable sites analyses in *Pennisetum* chloroplast gemomes.

	**Number of sites**	**Variable sites**		**Parsimony-informative sites**		**Nucleotide diversity**
		**Numbers**	**%**	**Numbers**	**%**	
LSC	82,523	1,474	1.79%	171	0.21%	0.00914
SSC	12,551	273	2.18%	37	0.29%	0.01111
IR	22,442	70	0.31%	8	0.04%	0.00161
Complete cp genome	139, 958	1,887	1.35%	224	0.16%	0.00683

**FIGURE 3:**
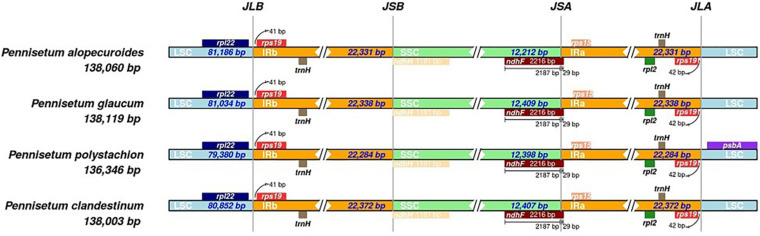
Comparison for border positions of LSC, SSC, and IR regions among four *Pennisetum*. Genes are denoted by boxes, and the gap between the genes and the boundaries is indicated by the number of bases unless the gene coincides with the boundary. Extensions of genes are also indicated above the boxes.

The bounder positions of chloroplast genomes of the four *Pennisetum* species were comprehensively compared. The four chloroplast genomes sequenced in this study have identical gene positions in the boundary regions (Figure 3).

### Sequence Divergence

**FIGURE 4:**
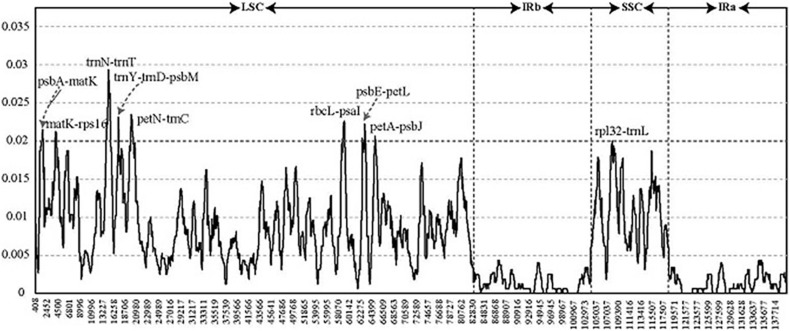
Comparative analysis of the nucleotide diversity values among four *Pennisetum* chloroplast genomes. *X*-axis: the position of the midpoint of a window (kb). *Y*-axis: nucleotide diversity of each window.

To detect highly variable regions, we analyzed variable sites in the *Pennisetum* chloroplast genome by sliding window analysis using the software DnaSP (Figure 4). Nine divergent loci (*psbA-matK*, *matK-rps16*, *trnN-trnT*, *trnY-trnD-psbM*, *petN-trnC*, *rbcL-psaI*, *psbE-petL*, *petA-psbJ*, and *rpl32-trnL*) had a Pi value greater than or equal to 0.02. All of these nine divergent loci were intergenic regions and were present in the LSC region, except for *rpl32-trnL*, which occurred in the SSC region, with none detected in the IR region. These results also confirmed that the LSC and SSC regions were less conserved than the IR regions.

We evaluated the marker divergence in this study by comparison with three accepted candidate DNA barcodes (*matK*, *rbcL*, and *psbA-trnH*). We found that the newly identified markers had higher variability than these DNA barcodes (Table 4). The highest variability was detected in the *rbcL-psaI* region (4.68%), followed by that in the *petN-trnC* (4.51%), *trnY-trnD-psbM* (3.87%), *trnN-trnT* (3.74%), and *psbE-petL* (3.72%) regions.

**TABLE 4:** Variability of nine novel markers and the three universal chloroplast DNA barcodes in *Pennisetum.*

**Markers**	**Length**	**Variable sites**		**Information sites**		**Nucleotide diversity**
		**Numbers**	**%**	**Numbers**	**%**	
*psbA-matK*	1,144	39	3.41	4	0.35	0.01652
*matK-rps16*	946	33	3.49	11	1.16	0.02019
*trnN-trnT*	2,511	94	3.74	6	0.24	0.02078
*trnY-trnD-psbM*	1,162	45	3.87	3	0.26	0.02313
*petN-trnC*	997	45	4.51	4	0.4	0.02354
*rbcL-psaI*	1,046	49	4.68	4	0.38	0.01917
*petA-psbJ*	1,424	45	3.16	2	0.14	0.01617
*psbE-petL*	834	31	3.72	4	0.48	0.02063
*rpl32-trnL*	887	32	3.61	4	0.45	0.02
*matK*	1,539	31	2.01	6	0.39	0.01076
*rbcL*	1,431	13	0.91	3	0.21	0.00489
*psbA-trnH*	544	7	1.29	2	0.37	0.00715

### SSR Analysis

**FIGURE 5:**
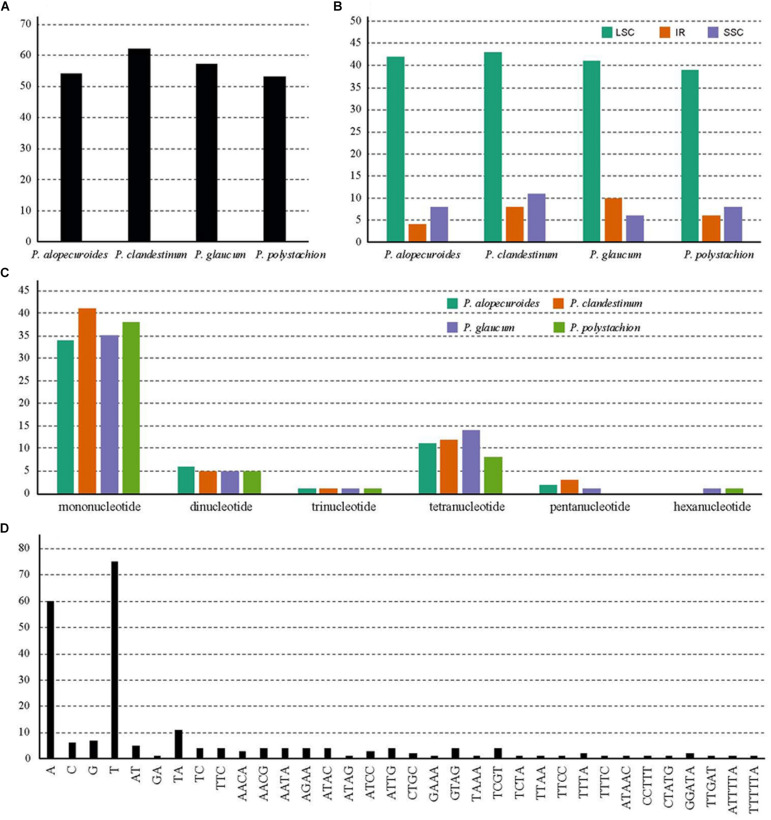
Analysis of simple sequence repeats (SSRs) in four Pennisetum chloroplast genomes. **(A)** The number of SSRs detected in the four chloroplast genomes; **(B)** The number of SSRs in large single-copy (LSC), inverted repeat (IR), and small single-copy (SSC) regions in the four chloroplast genomes; **(C)** The number of SSR types detected in the four sequenced chloroplast genomes; **(D)** The frequency of identified SSR motifs in different repeat class types.

A total of 226 SSRs were discovered in this study (Figure 5A). The chloroplast genomes of the four species harbored a similar number of SSRs (54, 62, 57, and 53). The number of SSRs was highest in *P. clandestinum* (62) and lowest in *P. polystachion* (53). Most of these SSRs were located in the LSC region (Figure 5B). The number of SSRs was similar between the IR and SSC regions. The numbers of mono-, di-, tri-, tetra-, penta-, and hexanucleotides were 148, 21, 4, 45, six, and two, respectively (Figure 5C). Additionally, pentanucleotide SSRs were found in three *Pennisetum* species, except for *P. polystachion*. Hexanucleotide SSRs were found in *P. glaucum* and *P. polystachion*. Mononucleotide repeats were the most abundant repeats, accounting for 65.5% of the total repeats, while tetranucleotide repeats accounted for 19.9%, and other SSRs accounted for 14.6%. Additionally, the majority of mononucleotide repeats belonged to A/T type (91.2%), and dinucleotide, trinucleotide, tetranucleotide, pentanucleotide, and hexanucleotide SSRs were especially rich in A or T (Figure 5D).

### Codon Usage

**FIGURE 6:**
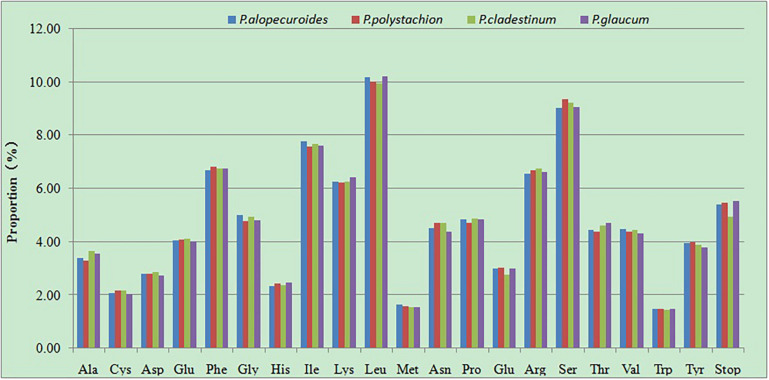
Amino acid proportion in four *Pennisetum* species protein-coding sequences.

The RSCU of the chloroplast genomes of four *Pennisetum*species was calculated using all protein-coding genes. These genes are encoded in 45,448–46,020 codons. Of the 45,448–46,020 codons, leucine (Leu) which encoded by UUA, UUG, CUU, CUC, CUA, and CUG was the most plentiful amino acid, with a frequency of 9.96–10.22% encoded by these codons, comprising 4,557–4,704 of the total number of codons, then serine (Ser) which encoded by UCU, UCC, UCA, UCG, AGU, and AGC was the second abundant amino acid, with a frequency of 9.04–9.36% (4,158–4,253 codons), while tryptophan (Trp) which encoded by UGG was the least frequently encoded amino acid, with a proportion of 1.45–1.5% (665–688 codons) (Figure 6 and Supplementary Table 2). The RSCU can eliminate the influence of amino acid composition on codon usage. Among the codons which the RSCU values are greater than one, the majority of the codons ended with A or U, On the contrary, the codons ended with C or G had RSCU values less than one. This result is consistent with previous studies (Lu et al., 2018; Huang et al., 2020).

### Phylogenetic Analysis

**FIGURE 7:**
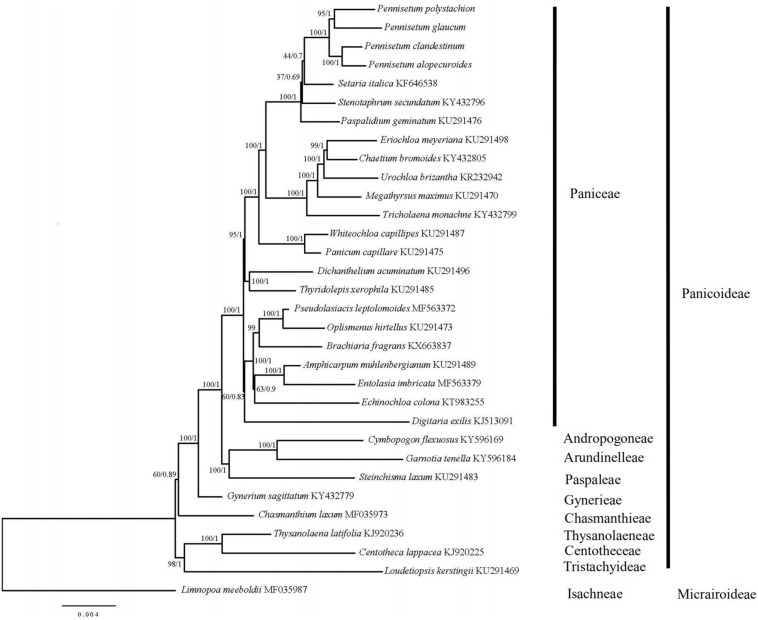
Phylogenetic tree constructed from 32 chloroplast genomes using ML and BI method. The bootstrap values were based on 1000 replicates and were indicated next to the branches.

In order to resolve the phylogenetic relationships of *Pennisetum*, the complete chloroplast genomes of 31 species in the Poaceae subfamily Panicoideae and one outgroup in the Poaceae subfamily Micrairoideae were used to construct phylogenetic trees (Figure 7). The ML tree and BI trees contained 19 of 29 nodes, with ML bootstrap values of 100%, and BI posterior probabilities of 1.0. Both the ML and BI phylogenetic analyses strongly demonstrated *Pennisetum* as a monophyletic group with 100% bootstrap support and 1.0 posterior probability. *Pennisetum polystachion* and *P. glaucum* clustered together with 95% bootstrap support and 1.0 posterior probability, and *P. clandestinum* and *P. alopecuroides* clustered together with 100% bootstrap support and 1.0 posterior probability. The clade formed by all four *Pennisetum* species was most closely related to the species *Setaria italica.*

## Discussion

### Chloroplast Sequence Variation

In the current study, the chloroplast genome sequences of four species of *Pennisetum* (*P. alopecuroides*, *P. clandestinum*, *P. glaucum*, and *P. polystachion*) were sequenced using Illumina sequencing and compared. The results showed that the size of the genome ranged from 136,346 (*P. polystachion*) to 138,119 bp (*P. glaucum*), which was longer than the genome of *S. italica* (Wang and Gao, 2016) and shorter than that of *Stenotaphrum secundatum* and *Paspalidium geminatum* (Burke et al., 2016). This length was within the size range of the chloroplast genomes of most angiosperms (Dong et al., 2013). Angiosperm chloroplast genomes usually contain 110–130 genes, with up to 80 protein-coding genes, four rRNA genes, and approximately 30 tRNA genes (Jansen and Ruhlman, 2012). In the current study, all four *Pennisetum* chloroplast genomes had 110 unique genes consisting of 76 protein-coding genes, four rRNA genes, and 30 tRNA genes. Comparative analysis of the *Pennisetum* chloroplast genomes using mVISTA revealed the DNA sequence similarities among related species. They were similar in structure, content, and order (Figure 1 and Table 1). The genome size and organization of the intergenic spacers were consistent with the observed variations in the size of the *Pennisetum* chloroplast genomes. Although the IR regions are more conserved than the SSC and LSC regions in the chloroplast genome, the evolutionary events and variation in the size of the chloroplast genomes in different plants could also be attributed to the expansion and contraction of the border between the SC and IR regions (Raubeson et al., 2007; Wang et al., 2008; Wang and Messing, 2011; Meng et al., 2018; Zhao et al., 2018; Androsiuk et al., 2020). In this study, the genes *rps19, ndhF*, *ndhH*, and *psbA* were located at the juncture of the LSC/IR_B_, IR_B_/SSC, SSC/IR_A_, and IR_A_/LSC borders. Similar to most angiosperms, IR boundaries were found within *ndhF* and *rps19* genes in the presented chloroplast genomes of *Pennisetum.*

### Molecular Markers

SSRs, known as microsatellites, are significant repetitive elements of the entire genome that consist of only one or a few tandemly repeated nucleotides. They are the same units with different unit numbers located in the homologous regions and play important roles in many applications, including species identification, population genetics, and phylogenetic studies (Yang et al., 2011; Jiao et al., 2012; Zhang et al., 2016a,b; Androsiuk et al., 2020). The SSRs in chloroplast genomes are usually distributed in intergenic regions (Zhao et al., 2015). In the SSR analysis, 226 SSR loci were found in the four *Pennisetum* chloroplast genomes (Figure 4). Most of these SSR loci were located in the LSC region, followed by the SSC and IR regions. The most abundant were mononucleotide repeats, which contributed to A/T richness. These results are consistent with most reported angiosperms (Cui et al., 2019; Gao et al., 2019; Li W. et al., 2019; Li Y. et al., 2019). Analysis of the SSRs identified in the chloroplast genome of *Pennisetum* indicated that the chloroplast genome is highly conserved among the four species. The results showed that the genes of the chloroplast genome were largely identical, whereas the intergenic regions were more variable. In general, the chloroplast genome SSRs of the four *Pennisetum* exhibited abundant variation and are thus likely useful for detecting polymorphisms at the intraspecific level as well as developing markers for *Pennisetum* species for future evolutionary and genetic diversity studies.

DNA barcoding is a molecular technique for characterizing species of organisms using a short, standardized DNA region. Since it was first proposed in 2003 (Hebert et al., 2003), it has been used for a wide range of purposes, including to identify species (Badotti et al., 2017), to discover new species (Zhao et al., 2011), to support food safety and authenticity of labeling by confirming identity or purity (Galimberti et al., 2012; Huxley-Jones et al., 2012), and to help identify candidate exemplar taxa for comprehensive phylogenetic studies (Hajibabaei et al., 2007). The cytochrome coxidase subunit I (*COI*) gene has been widely used in previous studies and exhibits a high ability to distinguish between animal species requirements (Hebert et al., 2003). In plants, many loci have been proposed as plant barcodes. The CBOL (Consortium for the Barcode of Life) Plant Working Group as well as previous studies have evaluated and recommended the use of the chloroplast-derived DNA barcode fragments *rbcL* and *matK* (Kress and Erickson, 2007; CBOL Plant Working Group, 2009; Kool et al., 2012). In addition, *trnH-psbA*, the internal spacer region of the chloroplast gene, and the ITS region of the nuclear genome were also investigated (Kress and Erickson, 2007; Chen et al., 2010; Bellanger et al., 2015). Despite the above, the molecular data for *Pennisetum* are highly limited. In the four *Pennisetum* species with completely sequenced chloroplast genomes, the genetic variation in the chloroplast regions of *matK*, *rbcL*, and *psbA-trnH*, which are widely used as DNA barcodes in plants, was lower than expected. However, genome-wide comparative analyses based on Pi value supported the identification of nine highly variable regions that could be utilized as a source of potential DNA barcodes for species identification and the reconstruction of phylogenetic relationships within this plant group: *psbA-matK*, *matK-rps16*, *trnN-trnT*, *trnY-trnD-psbM*, *petN-trnC*, *rbcL-psaI*, *psbE-petL*, *petA-psbJ*, and *rpl32-trnL*. Highly Pi values in the *psbE-petL*, *rpl32-trnL*, and *petA-psbJ* regions were also reported in other plants (Dong et al., 2012). The *petN-trnC* and *rbcL-psaI* regions have been used as molecular markers for phylogenetic analyses (Morris and Duvall, 2010; Iu Barkalov and Kozyrenko, 2014). Overall, these highly divergent regions presented abundant information for molecular marker development in plant identification and investigating the phylogenetic relationships of *Pennisetum*.

### Phylogenetic Analysis

Chloroplast genome sequences have been successfully used for phylogenetic studies of angiosperms (Jansen et al., 2007; Huang et al., 2014; Kim et al., 2015). The phylogenetic position of *Pennisetum* in Poaceae was inferred by analyzing the complete chloroplast genome and 76 genes shared among 32 species in Poaceae, for which the full chloroplast genome sequence had been generated and officially published in the database of the NCBI. The ML and BI trees exhibited similar phylogenetic topologies. The phylogenetic trees (ML and BI) demonstrated a significant relationship among Poaceae with high bootstrap values and posterior probabilities (Figure 6). The results showed that the main relationship was the same as other studies among the Family Poaceae (Robert et al., 2015; Soreng et al., 2015). Paniceae, Andropogoneae, Arundinelleae, Paspaleae, Gynerieae, Chasmanthieae, Centotheceae, and Tristachyideae that should placed in Panicoideae lineage clustered together [BP_(ML)_ = 100%, PP = 1.00]. The genus *Limnopoa* was found to be the earliest diverging lineage. Our research object genus *Pennisetum* form a high support clade as a paraphyletic group [BP_(ML)_ = 100%, PP = 1.00]. The clade formed by all four *Pennisetum* species is a sister to the species *Setaria italica.* And the genus *Pennisetum* and other 19 genus should be placed in the Paniceae lineage. In the genus *Pennisetum*, *P. polystachion*, and *P. glaucum* could be confidently assigned to one clade [BP_(ML)_ = 95%, PP = 1.00], while *P. clandestinum* and *P. alopecuroides* clustered together with high support rate [BP_(ML)_ = 100%, PP = 1.00]. The results are consistent with previous studies (Martel et al., 1997). Our study on the *Pennisetum* chloroplast genome provides valuable information on these four *Pennisetum* species and will facilitate future phylogenetic studies as well as other studies of *Pennisetum* species.

## Conclusion

In this study, we sequenced and compared the chloroplast genomes of four *Pennisetum* species. The chloroplast genomes of the four species were highly conserved with respect to gene orientation, gene content, and GC content. The goal of the present study was to expand our understanding of the diversity of the genus *Pennisetum* and identify reliable and effective DNA barcodes for *Pennisetum* species. Several non-coding regions or several intergenic spacer regions (*psbA-matK, matK-rps16, trnN-trnT, trnY-trnD-psbM, petN-trnC, rbcL-psaI, petA-psbJ, psbE-petL*, and *rpl32-trnL*) were found to be the most appropriate DNA barcodes for evolutionary and genetic diversity studies of *Pennisetum*. The molecular data in this study enhance the chloroplast genome resources for the family Poaceae and genus *Pennisetum* and could be useful for species identification, phylogenic analysis, and evolutionary history analysis.

## Data Availability Statement

The datasets presented in this study can be found in online repositories. The names of the repository/repositories and accession number(s) can be found below: https://www.ncbi.nlm.nih.gov/genbank/, MN180104; https://www.ncbi.nlm.nih.gov/genbank/, MW816925; https://www.ncbi.nlm.nih.gov/genbank/, MW816926; and https://www.ncbi.nlm.nih.gov/genbank/, MW816927.

## Author Contributions

JX designed the experiment and drafted the manuscript. CL collected the samples and performed the experiment. JX and YS analyzed the data. ML contributed reagents and analysis tools. All of the authors have read and approved the final manuscript.

## Conflict of Interest

The authors declare that the research was conducted in the absence of any commercial or financial relationships that could be construed as a potential conflict of interest.

## Publisher’s Note

All claims expressed in this article are solely those of the authors and do not necessarily represent those of their affiliated organizations, or those of the publisher, the editors and the reviewers. Any product that may be evaluated in this article, or claim that may be made by its manufacturer, is not guaranteed or endorsed by the publisher.

## Footnotes

1
http://www.ncbi.nlm.nih.gov/genome

2
https://tandem.bu.edu/trf/trf.html
