# Supplementary material for: Comparative Analysis of the Chloroplast Genome for Four Pennisetum Species: Molecular Structure and Phylogenetic Relationships
Source: Front Genet. 2021 Jul 27;12:687844. doi: 10.3389/fgene.2021.687844 (PMC8354216; doi:10.3389/fgene.2021.687844)
Supplement: Supplementary file 2 [file Table_2.doc]

**Table S2.** Codons in chloroplast genome of four *Pennisetum* species

| Amino acid | Codon | No.1 | No.2 | No.3 | No.4 | RSCU1 | RSCU2 | RSCU3 | RSCU4 | tRNA |
| --- | --- | --- | --- | --- | --- | --- | --- | --- | --- | --- |
| Phe | UUU | 1864 | 1848 | 1867 | 1921 | 1.21 | 1.19 | 1.20 | 1.24 |  |
| Phe | UUC | 1219 | 1254 | 1238 | 1183 | 0.79 | 0.81 | 0.8 | 0.76 | trnF-GAA |
| Leu | UUA | 1029 | 994 | 988 | 1057 | 1.32 | 1.31 | 1.29 | 1.35 | trnL-UAA |
| Leu | UUG | 905 | 900 | 868 | 883 | 1.16 | 1.18 | 1.14 | 1.13 | trnL-CAA |
| Leu | CUU | 980 | 957 | 984 | 987 | 1.26 | 1.26 | 1.29 | 1.26 |  |
| Leu | CUC | 575 | 581 | 581 | 555 | 0.74 | 0.76 | 0.76 | 0.71 |  |
| Leu | CUA | 786 | 725 | 753 | 796 | 1.01 | 0.95 | 0.99 | 1.02 | trnL-UAG |
| Leu | CUG | 408 | 400 | 408 | 426 | 0.52 | 0.53 | 0.53 | 0.54 |  |
| Ile | AUU | 1519 | 1438 | 1496 | 1464 | 1.27 | 1.25 | 1.27 | 1.25 |  |
| Ile | AUC | 846 | 830 | 863 | 825 | 0.71 | 0.72 | 0.73 | 0.71 | trnI-GAU |
| Ile | AUA | 1220 | 1177 | 1177 | 1218 | 1.02 | 1.02 | 1.00 | 1.04 | trnI-CAU |
| Met | AUG | 754 | 712 | 715 | 719 | 1.00 | 1.00 | 1.00 | 1.00 | trn(f)M-CAU |
| Val | GUU | 687 | 621 | 705 | 654 | 1.33 | 1.25 | 1.38 | 1.32 |  |
| Val | GUC | 390 | 367 | 347 | 352 | 0.75 | 0.74 | 0.68 | 0.71 | trnV-GAC |
| Val | GUA | 629 | 613 | 652 | 616 | 1.22 | 1.23 | 1.27 | 1.24 | trnV-UAC |
| Val | GUG | 363 | 387 | 346 | 367 | 0.70 | 0.78 | 0.68 | 0.74 |  |
| Ser | UCU | 1030 | 1026 | 1037 | 1024 | 1.49 | 1.45 | 1.46 | 1.47 |  |
| Ser | UCC | 828 | 844 | 797 | 807 | 1.19 | 1.19 | 1.12 | 1.16 | trnS-GGA |
| Ser | UCA | 722 | 730 | 742 | 751 | 1.04 | 1.03 | 1.05 | 1.08 | trnS-UGA |
| Ser | UCG | 473 | 533 | 530 | 564 | 0.68 | 0.75 | 0.75 | 0.891 |  |
| Pro | CCU | 586 | 580 | 649 | 650 | 1.05 | 1.08 | 1.16 | 1.16 |  |
| Pro | CCC | 568 | 524 | 560 | 543 | 1.02 | 0.98 | 1.00 | 0.97 |  |
| Pro | CCA | 732 | 702 | 702 | 681 | 1.32 | 1.31 | 1.26 | 1.22 | trnP-UGG |
| Pro | CCG | 339 | 334 | 326 | 364 | 0.61 | 0.62 | 0.58 | 0.65 |  |
| Thr | ACU | 633 | 612 | 656 | 658 | 1.23 | 1.23 | 1.24 | 1.22 |  |
| Thr | ACC | 547 | 515 | 547 | 562 | 1.07 | 1.03 | 1.03 | 1.04 | trnT-GGU |
| Thr | ACA | 527 | 544 | 582 | 594 | 1.03 | 1.09 | 1.10 | 1.10 | trnT-UGU |
| Thr | ACG | 347 | 321 | 339 | 350 | 0.68 | 0.64 | 0.64 | 0.65 |  |
| Ala | GCU | 507 | 473 | 530 | 548 | 1.29 | 1.26 | 1.26 | 1.34 |  |
| Ala | GCC | 348 | 335 | 353 | 333 | 0.89 | 0.89 | 0.84 | 0.81 |  |
| Ala | GCA | 442 | 420 | 510 | 474 | 1.13 | 1.12 | 1.22 | 1.16 | trnA-UGC |
| Ala | GCG | 272 | 273 | 283 | 286 | 0.69 | 0.73 | 0.68 | 0.70 |  |
| Tyr | UAU | 1178 | 1212 | 1214 | 1169 | 1.30 | 1.34 | 1.36 | 1.35 |  |
| Tyr | UAC | 638 | 594 | 568 | 569 | 0.70 | 0.66 | 0.64 | 0.65 | trnY-GUA |
| Stop | UAA | 1003 | 959 | 884 | 1008 | 1.21 | 1.15 | 1.16 | 1.19 |  |
| Stop | UAG | 779 | 825 | 701 | 817 | 0.94 | 0.99 | 0.92 | 0.96 |  |
| His | CAU | 735 | 768 | 772 | 792 | 1.37 | 1.38 | 1.42 | 1.39 |  |
| His | CAC | 339 | 347 | 313 | 350 | 0.63 | 0.62 | 0.58 | 0.61 | trnH-GUG |
| Gln | CAA | 949 | 939 | 893 | 947 | 1.38 | 1.37 | 1.40 | 1.37 | trnQ-UUG |
| Gln | CAG | 425 | 435 | 381 | 436 | 0.62 | 0.63 | 0.60 | 0.63 |  |
| Asn | AAU | 1417 | 1418 | 1513 | 1398 | 1.36 | 1.32 | 1.40 | 1.38 |  |
| Asn | AAC | 664 | 724 | 656 | 626 | 0.64 | 0.68 | 0.60 | 0.62 | trnN-GUU |
| Lys | AAA | 1879 | 1838 | 1924 | 1885 | 1.31 | 1.30 | 1.34 | 1.28 | trnK-UUU |
| Lys | AAG | 998 | 989 | 950 | 1067 | 0.69 | 0.70 | 0.66 | 0.72 |  |
| Asp | GAU | 889 | 864 | 921 | 888 | 1.37 | 1.36 | 1.40 | 1.41 |  |
| Asp | GAC | 405 | 403 | 393 | 371 | 0.63 | 0.64 | 0.60 | 0.59 | trnD-GUC |
| Glu | GAA | 1277 | 1246 | 1276 | 1262 | 1.37 | 1.34 | 1.35 | 1.36 | trnE-UUC |
| Glu | GAG | 590 | 609 | 614 | 595 | 0.63 | 0.66 | 0.65 | 0.64 |  |
| Cys | UGU | 551 | 556 | 565 | 535 | 1.15 | 1.12 | 1.13 | 1.14 |  |
| Cys | UGC | 409 | 433 | 435 | 403 | 0.85 | 0.88 | 0.87 | 0.86 | trnC-GCA |
| Stop | UGA | 705 | 707 | 697 | 719 | 0.85 | 0.85 | 0.92 | 0.85 |  |
| Trp | UGG | 688 | 679 | 665 | 682 | 1.00 | 1.00 | 1.00 | 1.00 | trnW-CCA |
| Arg | CGU | 346 | 327 | 378 | 374 | 0.69 | 0.65 | 0.73 | 0.74 | trnR-ACG |
| Arg | CGC | 272 | 245 | 222 | 221 | 0.54 | 0.48 | 0.43 | 0.44 |  |
| Arg | CGA | 494 | 527 | 525 | 523 | 0.98 | 1.04 | 1.01 | 1.03 |  |
| Arg | CGG | 311 | 326 | 299 | 329 | 0.62 | 0.64 | 0.58 | 0.65 |  |
| Arg | AGA | 992 | 959 | 1046 | 980 | 1.97 | 1.90 | 2.02 | 1.93 | trnR-UCU |
| Arg | AGG | 600 | 651 | 640 | 619 | 1.19 | 1.29 | 1.23 | 1.22 |  |
| Ser | AGU | 630 | 632 | 648 | 616 | 0.91 | 0.89 | 0.91 | 0.88 |  |
| Ser | AGC | 475 | 491 | 499 | 418 | 0.69 | 0.69 | 0.70 | 0.60 | trnS-GCU |
| Gly | GGU | 548 | 508 | 553 | 507 | 0.95 | 0.93 | 0.97 | 0.91 |  |
| Gly | GGC | 332 | 335 | 343 | 320 | 0.58 | 0.62 | 0.60 | 0.58 | trnG-GCC |
| Gly | GGA | 828 | 756 | 805 | 820 | 1.44 | 1.39 | 1.41 | 1.48 | trnG-UCC |
| Gly | GGG | 599 | 576 | 577 | 576 | 1.04 | 1.06 | 1.01 | 1.04 |  |
